# Primiparas’ prenatal depressive symptoms, anxiety, and salivary oxytocin level predict early postnatal maternal–infant bonding: a Japanese longitudinal study

**DOI:** 10.1007/s00737-024-01441-5

**Published:** 2024-02-26

**Authors:** Hitomi Kanekasu, Yachiyo Shiraiwa, Shu Taira, Hiroko Watanabe

**Affiliations:** 1 Department of Children and Women’s Health, Osaka University Graduate School of Medicine, Suita, Osaka 565-0871 Japan; 2 Adachi Hospital, Kyoto, Kyoto, 604-0837 Japan; 3 Faculty of Food and Agricultural Sciences, Fukushima University, Kanayagawa, Fukushima 960-1248 Japan

**Keywords:** Depressive symptoms, Anxiety, Oxytocin, Maternal–infant bonding

## Abstract

**Purpose:**

It was reported that maternal-infant bonding failure predicts abusive parenting. Maternal-infant bonding is important to prevent child abuse. This study aimed to investigate the association between prenatal depressive symptoms, anxiety, cortisol, and oxytocin levels, and postnatal maternal–infant bonding.

**Methods:**

The participants completed a self-report prenatal questionnaire that included the Edinburgh Postnatal Depression Scale (EPDS) and State-Trait Anxiety Inventory (STAI) in the second trimester. Blood and saliva were collected in the second trimester. Cortisol levels were measured in plasma, while oxytocin levels were measured in saliva. Postnatal questionnaires, including the Mother-to-Infant Bonding Scale (MIBS), were administered at 2–5 days, 1 month, and 3 months postpartum. Multiple linear regression and generalized estimating equation (GEE) were conducted for analysis.

**Results:**

Sixty-six primiparas participated in the study. Prenatal depressive symptoms (EPDS ≥ 9) and anxiety (STAI-S ≥ 42) were observed in 21.2% and 28.8% of the participants, respectively. The median cortisol and oxytocin levels were 21.0 µg/dL and 30.4 pg/mL, respectively. Multivariate linear regression showed that postnatal social support, prenatal depressive symptoms, anxiety, and salivary oxytocin levels predicted MIBS scores at 2–5 days postpartum. At 1 month postpartum, household income, history of miscarriage, postnatal social support, and prenatal anxiety predicted MIBS scores. At 3 months postpartum, only postnatal social support predicted MIBS scores. The results of GEE showed that prenatal anxiety, oxytocin levels, postpartum period, household income, and postpartum social support were associated with MIBS scores.

**Conclusion:**

Prenatal depressive symptoms, anxiety, and lower salivary oxytocin levels were predicted to worsen maternal–infant bonding at 2–5 days postpartum. Prenatal anxiety was predicted to cause the same 1 month postpartum. Measuring prenatal depressive symptoms, anxiety, and salivary oxytocin levels may render the assessment of the risk of maternal–infant bonding failure during the early postpartum period and intervene during pregnancy possible.

## Introduction

Maternal–infant relationship dysfunction has been reported as an important perinatal mental health issue (Brockington 2004) and is termed bonding failure or disorder. Some mothers experience feelings of rejection or anger toward their children during the postpartum period (Brockington et al. 2006). Maternal–infant bonding has been reported to affect parenting behavior (Kinsey et al. 2013). Kitamura et al. (2013) reported that bonding failure predicts abusive parenting at 3 months postpartum. Ohashi et al. (2016) presented that early postpartum maternal bonding failure predicts neonatal emotional abuse. Maternal-infant bonding failure appears to be associated with abusive parenting and child abuse. Hence, improvements in maternal–infant bonding failure should prevent child abuse.

A scoping review of maternal–infant bonding (Edwards et al. 2017) reported many risk factors for maternal–infant bonding, including maternal mental health, attitude toward the infant, lifestyle, and obstetric history. Furthermore, the relationship between maternal–infant bonding and social support (Badr et al. 2018; Ohara et al. 2018) and partner violence (Kita et al. 2016; Nishigoori et al. 2019) have been investigated. Especially, many studies have investigated the relationships between perinatal depressive symptoms, anxiety, and maternal–infant bonding. However, a number of these are either cross-sectional studies (Afolabi et al. 2020; Lutkiewicz et al. 2020; Tikotzky 2016) or have investigated the relationships between depressive symptoms, anxiety, and maternal–infant bonding only in the postpartum period (Kerstis et al. 2016; Motegi et al. 2020; Rossen et al. 2019). In order to prevent the maternal-infant bonding failure, it is important to confirm whether there are any risk factors during pregnancy. For prenatal screening of the risk of bonding failure, it is necessary to investigate the association between prenatal depressive symptoms, anxiety, and postnatal maternal–infant bonding.

Previous studies have investigated the association between cortisol, oxytocin, and depressive symptoms in the perinatal period. A systematic review has suggested that hypercortisolemia is linked to transient depressive states, whereas hypocortisolemia is associated with chronic depression (Seth et al. 2016). Cortisol is a glucocorticoid produced by the hypothalamic–pituitary–adrenal axis (Levine et al. 2018). It has long been used as a biological marker for stress, anxiety, and depression. Few studies have investigated the relationship between maternal cortisol and postpartum maternal-infant bonding. However, cortisol may also predict postpartum bonding, as it has been reported that there is a relationship between stress, anxiety, depressive symptoms and maternal-infant bonding (Lutkiewicz et al. 2020). Similarly, a review reported that mothers with higher oxytocin levels presented fewer depressive symptoms than mothers with lower oxytocin levels (Moura et al. 2016). Oxytocin is an important peptide hormone synthesized in the hypothalamus during birth and lactation. Oxytocin is associated with various conditions and diseases, including mental stress, anxiety, and mood disorders (McDonald and McDonald 2010). Additionally, oxytocin is known to be an agent in the calm and connection responses (Uvnäs-Moberg et al. 2005). Therefore, these hormones may be useful biomarkers for prenatal depressive symptoms and anxiety.

Therefore, our study aimed to investigate the association between prenatal depressive symptoms, anxiety, cortisol, oxytocin, and postnatal maternal–infant bonding, in addition to conventional risk factors. We hypothesized that prenatal depressive symptoms, anxiety, higher cortisol, and lower oxytocin levels could predict worsening maternal–infant bonding at 25 days, 1 month, and 3 months postpartum.

## Methods

### Participants and procedure

The participants in this longitudinal observational study were recruited in the second trimester between April 2018 and September 2019. The research hospital is a secondary medical facility in Kyoto, Japan. Only primiparous women were included in this study because factors related to older children may affect maternal–infant bonding. The inclusion criteria encompassed individuals meeting the following conditions: (a) primiparous, (b) experiencing a singleton pregnancy, (c) aged between 20 and 40 years, and (d) of Japanese ethnicity. The exclusion criteria comprised individuals with the following condition: (a) chronic diseases such as essential hypertension and type 1 or type 2 diabetes, (b) mental diseases 66 such as schizophrenia, and (c) fetal disorders. Saliva and blood samples were collected during the second trimester at a research hospital. Participants completed self-report questionnaires in the second trimester, 2–5 days postpartum, 1 month, and 3 months postpartum. These questionnaires were distributed to participants at their prenatal and postnatal checkups. The prenatal questionnaire was used to assess maternal information, depressive symptoms, and anxiety levels. The postnatal questionnaire assessed postnatal social support and maternal–infant bonding. Other perinatal information was obtained from participants’ medical records by the researcher.

## Measures and instruments

### Demographic and perinatal variables

The demographic and perinatal variables were selected based on a scoping review (Edwards et al. 2017) and previous studies (Nakano et al. 2019; Ohara et al. 2018). Maternal information included maternal demographic data, such as maternal education, marital status, and household income. Perinatal information included pregnancy, intrapartum, and postpartum data, such as infertility treatment, mode of delivery, infant sex, breastfeeding, and postpartum social support. Data on breastfeeding and postpartum social support were collected at 1 month postpartum. Postpartum social support was measured using the Multidimensional Scale of Perceived Social Support (MSPSS) (Japanese version) developed by Zimet et al. (1988). Iwasa et al. (2007) also reported high validity and reliability. The MSPSS consists of 12 items rated on a 7-point Likert scale. The mean MSPSS score was used as social support score. In the present study, Cronbach’s α for the MSPSS was 0.93.

### Depressive symptoms

Depressive symptoms were assessed using the Edinburgh Postnatal Depression Scale (EPDS). The EPDS is a reliable questionnaire that has been used to measure depressive symptoms during prenatal and postnatal periods (Cox and Holden 1987; Murray and Cox 1990; Bennett et al. 2004). The EPDS comprises 10 items with scores ranging from 0–3, and the total score ranges from 0–30. It was reported that the sensitivity and specificity were 75% and 93%, respectively, in the Japanese version of the EPDS using a cut-off point of 8/9 (Okano et al. 1996). This cutoff point has been commonly used in Japanese prenatal and postnatal studies (Nakamura et al. 2020; Ohara et al. 2018; Takehara et al. 2018). Cronbach’s α for the EPDS in this study was 0.72.

### Anxiety

The State-Trait Anxiety Inventory (STAI) assesses state and trait anxiety (Spielberger et al. 1971). State anxiety, which indicated temporary anxiety at the time of assessment, was used for this study. State anxiety consisted of 20 items rated on a 4-point Likert scale ranging from 1–4. The scores range from 20–80, with higher scores indicating higher state anxiety. The Japanese version of the STAI has a high validity and reliability (Nakazato and Mizuguti 1982). For women, a cut-off score of 41/42 for state anxiety was adopted in previous Japanese studies (Koyama et al. 2016; Yamanishi et al. 2013). Cronbach’s α for state anxiety was 0.91 in the present study.

### Maternal–infant bonding

Maternal–infant bonding was measured using the Japanese version of the Mother-to-Infant Bonding Scale (MIBS), developed by Yoshida et al. (2012). The MIBS consists of 10 items rated on a 4-point Likert scale ranging from 0–3. Higher scores indicated worse maternal–infant bonding. Adoptable validity and reliability have been reported (Yoshida et al. 2012). The MIBS is commonly used to assess maternal–infant bonding in Japan (Kita et al. 2016; Kitamuta et al. 2015; Motegi et al. 2020). In the present study, Cronbach’s α for the MIBS was 0.634 at 2–5 days postpartum, 0.77 at 1 month postpartum, and 0.85 at 3 months postpartum.

### Cortisol

Maternal blood samples were collected between 9:00–14:00 as part of routine blood tests during the second trimester. Blood samples were transported to the laboratory within 1 day (Japan Clinical Laboratories, Inc., Kyoto, Japan). Cortisol levels were assayed by electrochemiluminescence immunoassay. Intra-assay and inter-assay coefficient variations were < 20% and < 15%, respectively.

### Oxytocin

Maternal saliva samples were collected using a saliva collection aid (Salimetrics, LLC, Carlsbad, CA) in a private outpatient room after a routine check-up. The saliva collection aids and cryovials were kept sufficiently chilled on ice before collection. Participants were asked not to eat or drink for 30–60 min before sample collection. At least 1.0 mL of saliva was collected using a passive drool. Saliva samples were collected between 9:00–14:00. After collection, saliva samples were stored at -80 °C in the laboratory. Saliva samples were extracted four times (Carter et al. 2007). Oxytocin levels were assayed in duplicates using commercial enzyme-linked immunosorbent assay kits (ENZO Life Sciences, Ann Arbor, MI). according to the manufacturer’s instructions. The product manual reported that the intra- and inter-assay coefficients of variability were 12.6–13.3% and 11.9–20.9%, respectively. In the present study, the intra-assay coefficient variation was ≤ 19.8%.

### Statistical analysis

For missing MIBS scores at 1 and 3 months postpartum, the last observation was conducted in each of the three cases (No.41, 105, 124). Log transformation was performed to determine the plasma cortisol and salivary oxytocin levels. Descriptive statistics were used to identify the demographic characteristics, salivary oxytocin and plasma cortisol levels, state anxiety, EPDS, and MIBS scores. Normal distributions of continuous variables including log-transformed oxytocin and cortisol values were confirmed using the Shapiro–Wilk test. The Chi-square, Fisher's exact, independent *t*-, and Mann–Whitney *U* tests were used to compare participants and dropouts on demographic characteristics and mother-infant bonding. The Friedman test, Wilcoxon signed-rank test, and Bonferroni correction were used to compare MIBS scores at 2–5 days, 1 month, and 3 months postpartum.

Multiple linear regression analyses were conducted to examine the relationships between demographic and perinatal variables, prenatal mental health variables, and MIBS scores in each postpartum period. The demographic and perinatal variables were obtained from previous studies (Edwards et al. 2017; Nakano et al. 2019; Ohara et al. 2018). We set prenatal anxiety and depressive symptoms, salivary oxytocin, and plasma cortisol levels in the models as prenatal mental health variables. As cortisol and oxytocin levels are influenced by diurnal rhythms (Eriksson et al. 1989; Lindow et al. 1996), the models were adjusted for saliva and blood sampling times. Categorical variables were coded as dummy variables. A stepwise variable selection was used for the models. We confirmed the multicollinearity and normal distribution of the residuals.

Finally, we used generalized estimating equations (GEE) to examine the association between MIBS scores from 2–5 days to 3 months postpartum, and independent variables. Independent variables included demographic and perinatal variables that were associated in multiple regression analyses, and prenatal mental health variables.

A *P* value of less than 0.05 was considered statistically significant. All statistical analyses were performed using SPSS for Windows (version 24.0; IBM, Armonk, NY, USA).

## Results

### Participant characteristics

**Fig. 1:**
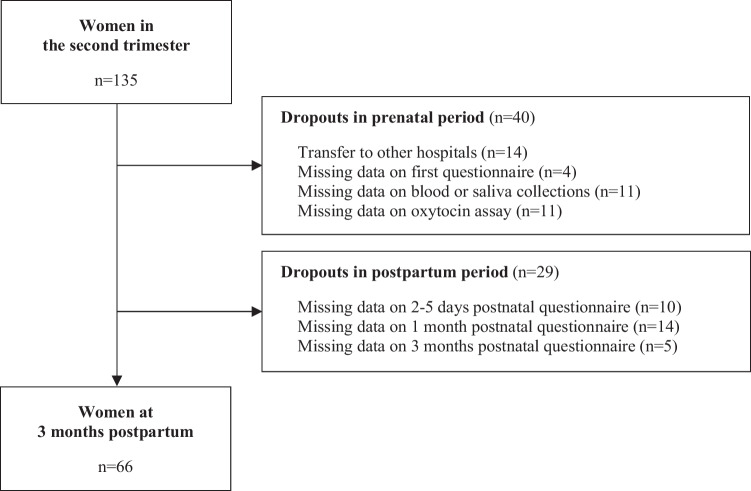
Flowchart for the study sample

We estimated an effect size of 0.64 based on the results of Fransson et.al. (2011). We set the effect size = 0.64, α = 0.05, power = 0.8, calculated the sample size using a two-tailed test. We recruited 135 primiparas anticipating a dropout rate of 20%, with a final sample size of 106. Among them, 14 participants were transferred to other hospitals during pregnancy, 4 participants did not complete the first questionnaire, and 11 participants did not undergo blood or saliva collection. Oxytocin assays were not performed correctly in 11 samples. These samples were considered to have precision issues, because of an intra-assay coefficient of oxytocin of 20% or higher. Furthermore, 29 participants did not complete the postnatal questionnaires. Therefore, it was not possible to collect all data for 69 primiparas (dropout rate 51.1%). Finally, 66 participants were included in the analysis (Fig. 1). The saliva samples were collected at 23.0 ± 1.6 weeks of gestational age, and the blood samples were collected at 26.4 ± 1.4 weeks. The first questionnaire was conducted at 24.2 ± 1.9 weeks of gestational age, and the postnatal questionnaires were conducted at 3.12 ± 0.7 days postpartum, 4.8 ± 1.0 weeks postpartum, and 14.3 ± 2.0 weeks postpartum.

There were no differences in demographic characteristics or maternal–infant bonding between participants and dropouts (Table 1). The mean maternal age was 31.8 ± 3.8 years; 53.0% were university graduates, 95.3% were married, and 7.6% had unplanned pregnancies. Participants who had a history of miscarriage comprised 22.7%, and infertility treatment were 25.8%. The median gestational age was 39.9 (38.8, 40.4) weeks, and the mean birth weight was 3044.3 ± 341.7 g. Furthermore, 74.2% of infants were born by vaginal delivery (50.0% were without labor stimulants and 24.2% were with labor stimulants), 62.1% were male, and 30.3% were fed only breast milk in 1 month postpartum. The preterm birth was 4.5% and low birth weight was 6.1% in the present study.

**Table 1:** Demographic characteristics and maternal-infant bonding of included and excluded primiparas

	Participants (n = 66)	Dropouts (n = 50)	*p*
	N (%) / Median (IQR) / Mean ± SD	N (%) / Median (IQR) / Mean ± SD	
Age (year) ^c^	31.8 ± 3.8	32.7 ± 4.1	0.21
Education ^b^			0.96
Secondary or High school	8 (12.1)	5 (10.0)	
Junior or Technical college	19 (28.8)	13 (26.0)	
University	35 (53.0)	29 (58.0)	
Graduate school	4 (6.1)	3 (6.0)	
Marital status^b^			0.63
Married	63 (95.5)	49(98.0)	
Single	3 (4.5)	1 (2.0)	
Household income/year (JPY) ^b^			0.34
< 2 million	1 (1.5)	3 (6.0)	
2–5.9 million	28 (59.1)	25 (50.0)	
≥ 6 million	26 (39.4)	22 (44.0)	
History of miscarriage ^a^			0.87
Yes	15 (22.7)	12 (24.0)	
No	51 (77.3)	38 (76.0)	
Unplanned pregnancy ^b^			1.00
Yes	5 (7.6)	3 (6.0)	
No	61 (92.4)	47 (94.0)	
Infertility treatment ^a^			0.98
Yes	17 (25.8)	13 (26.0)	
No	49 (74.2)	37 (74.0)	
Mode of delivery ^a^			0.29
Vaginal delivery	49 (74.2)	36 (65.5)	
C-section	17 (25.8)	19 (34.5)	
Gestational age (week) ^d^	39.9 (38.8, 40.4)	39.6 (38.6, 40.4)	0.67
Infant birth weight (g) ^c^	3044.3 ± 341.7	3079.0 ± 370.1	0.60
Infant sex ^a^			0.13
Male	41 (62.1)	24 (48.0)	
Female	25 (37.9)	26 (52.0)	
Preterm birth (< 37 weeks) ^b^			1.00
Yes	3 (4.5)	2 (4.0)	
No	63 (95.5)	48 (96.0)	
Low birth weight (< 2500 g) ^b^			0.72
Yes	4 (6.1)	4 (8.0)	
No	62 (93.9)	46 (92.0)	
Breast feeding ^b^			0.28
Breast milk only	19 (28.8)	11 (22.0)	
Breast milk and formula	47 (71.2)	37 (74.0)	
Formula only	0 (0.0)	2 (4.0)	

a = Chi-square test, b = Fisher's exact test, c = Unpaired *t*-test, d = Mann–Whitney *U* test

### Maternal prenatal depressive symptoms, anxiety, plasma cortisol levels, and salivary oxytocin levels

Maternal prenatal depressive symptoms, anxiety, plasma cortisol, and salivary oxytocin levels in the second trimester are shown in Table 2. EPDS ≥ 9 was observed in 21.2%, and state anxiety ≥ 42 was observed in 28.8% of the participants. The median cortisol level was 21.0 (16.6, 24.1) µg/dL, and the median oxytocin levels was 30.4 (19.2, 114.2) pg/mL.

**Table 2:** Maternal prenatal anxiety, depressive symptoms, salivary oxytocin levels, and plasma cortisol levels in the second trimester

n = 66	
	N (%) / Median (IQR)
Depressive symptoms	
EPDS ≤ 8	52 (78.8)
EPDS ≥ 9	14 (21.2)
Anxiety	
State anxiety ≤ 41	47 (71.2)
State anxiety ≥ 42	19 (28.8)
Plasma cortisol level (µg/dL)	21.0 (16.6, 24.1)
Salivary oxytocin level (pg/mL)	30.4 (19.2, 114.2)

### MIBS scores in 2–5 days, 1 month, and 3 months postpartum

Table 3 shows the MIBS scores at 2–5 days, 1 month, and 3 months postpartum. The median MIBS scores at 2–5 days postpartum were 2.00 (1.00, 3.00), 1 month postpartum were 1.00 (0.00, 3.00), and 3 months postpartum were 0.00 (0.00, 2.00). There was a significant difference in the MIBS scores among the three periods (*p* = 0.000, *χ*^*2*^ = 34.75, *df* = *2*). Post hoc comparisons showed that the MIBS scores at 2–5 days postpartum were higher than those at 3 months postpartum (*adj. p* = 0.000). Similarly, the MIBS scores at 1 month postpartum were higher than those at 3 months postpartum (*adj. p* = 0.002). There was no significant difference between 2–5 days and 1 month postpartum (*adj. p* = 0.453).

**Table 3:** Comparison of MIBS scores in 2–5 days, 1 month, and 3 months postpartum

n = 66				
		MIBS scores	*p*	*χ*^*2*^
		Median (IQR)		
2–5 days postpartum		2.00 (1.00, 3.00)	0.000	34.75
1 month postpartum		1.00 (0.00, 3.00)		
3 months postpartum		0.00 (0.00, 2.00)		
Post hoc comparisons			*adj.p*	
MIBS scores				
2–5 days postpartum	-	1 month postpartum	0.453	
2–5 days postpartum	-	3 months postpartum	0.000	
1 month postpartum	-	3 months postpartum	0.002	

Friedman test, Bonferroni correction

### Maternal demographic characteristics variables, prenatal mental health, and MIBS scores

The results of the multivariate linear regression analysis examining the associations between demographic characteristics, prenatal mental health, and MIBS scores are shown in Table 4. In 2–5 days postpartum, four variables predicted the MIBS scores: postnatal social support, prenatal depressive symptoms, anxiety, and oxytocin levels (*R*^*2*^ = 0.410, *adj. R*^*2*^ = 0.350). At 1 month postpartum, four variables were associated with MIBS scores: household income, history of miscarriage, postnatal social support, and prenatal anxiety (*R*^*2*^ = 0.424, *adj. R*^*2*^ = 0.366). At 3 months postpartum, only postnatal social support was related to MIBS scores (*R*^*2*^ = 0.169, *adj. R*^*2*^ = 0.129). The variance inflation factors of the models were 1.02–1.50.

**Table 4:** Multivariable beta estimates for associations between demographic and perinatal variables, prenatal mental health, and MIBS scores

n = 66												
	2–5 days Postpartum				1 month postpartum				3 months postpartum			
	*β*	B	95%CI		*β*	B	95%CI		*β*	B	95%CI	
Demographic and perinatal variables												
Household income	-	-	-	-	-0.27**	-1.29	-2.25	-0.33	-	-	-	-
History of miscarriage	-	-	-	-	-0.26*	-1.54	-2.78	-0.31	-	-	-	-
Postnatal social support	-0.24*	-0.73	-1.39	-0.08	-0.40**	-1.34	-2.09	-0.60	-0.40**	-0.76	-1.20	-0.31
Prenatal mental health variables												
Prenatal depressive symptoms	0.28*	1.50	0.36	2.63	-	-	-	-	-	-	-	-
Prenatal anxiety	0.36**	1.78	0.66	2.89	0.41**	2.19	0.94	3.44	-	-	-	-
Prenatal oxytocin level	-0.23*	-1.26	-2.39	-0.14	-	-	-	-	-	-	-	-
*R*^*2*^	0.410				0.424				0.169			
*adj. R*^*2*^	0.350				0.366				0.129			

Models adjusted for saliva sampling time, and blood sampling time

Stepwise variable selection was used in models

Demographic and perinatal variables: education, marital status, household income, history of miscarriage, unplanned pregnancy, infertility treatment, mode of delivery, infant sex, breast feeding, postnatal social support (MPSS Scores)

Prenatal mental health variables: depressive symptoms (EPDS ≥ 9), anxiety (state anxiety ≥ 42), salivary oxytocin level and plasma cortisol level

* p < 0.05, ** p < 0.01, *** p < 0.001

The results of GEE are shown in Table 5. There was no difference at 1 month postpartum compared to 2–5 days postpartum (*p* = 0.314). However, a difference was found at 3 months postpartum (*p* = 0.000). As demographic and perinatal variables, household income (*p* = 0.004) and postpartum social support (*p* = 0.004) were associated with MIBS scores. Prenatal anxiety (*p* = 0.000) and oxytocin levels (*p* = 0.026) predicted MIBS scores as prenatal mental health variables.

**Table 5:** The results of generalized estimation equation for MIBS scores

n = 66				
	B	95%CI		*p*
Postpartum Period				
2–5 days postpartum	Reference	-	-	-
1 month postpartum	-0.32	-0.94	0.30	0.314
3 months postpartum	-1.50	-2.07	-0.93	0.000
Demographic and perinatal variables				
Household income	-0.74	-1.30	-0.24	0.004
History of miscarriage	-0.47	-1.14	0.20	0.168
Postnatal social support	-0.74	-1.18	-0.29	0.001
Prenatal mental health variables				
Prenatal depressive symptoms	0.47	-0.14	1.08	0.134
Prenatal anxiety	1.24	0.56	1.93	0.000
Prenatal cortisol level	-1.36	-4.09	1.37	0.330
Prenatal oxytocin level	-0.79	-1.49	-0.10	0.026

Models adjusted for saliva sampling time, and blood sampling time

## Discussion

Our study aimed to examine the association between prenatal depressive symptoms, anxiety, cortisol, oxytocin, and postnatal maternal–infant bonding. Prenatal depressive symptoms, anxiety, and salivary oxytocin levels were related to maternal–infant bonding at 2–5 days postpartum. Additionally, prenatal anxiety was predicted at 1 month postpartum. However, cortisol levels were not associated with postnatal bonding at 2–5 days, 1 month, or 3 months postpartum.

The median MIBS score was higher at 2–5 days and 1 month postpartum and declined at 3 months postpartum. Additionally, no difference was found between 2–5 days and 1 month postpartum, however a difference was found between 2–5 days and 3 months postpartum in the results of GEE. In other words, maternal–infant bonding was lower until 1 month postpartum and improved at 3 months postpartum. Holding the baby, breastfeeding, and rooming-in during the immediate postpartum period have been reported to promote postnatal maternal–infant bonding (Kinsey et al. 2013). It is possible that these child-rearing experiences naturally improve maternal–infant bonding. Similarly, Kinsey et al. (2014) reported that the lowest primipara bonding occurred at 1 month postpartum and the highest at 6 months postpartum.

In our study, prenatal depressive symptoms predicted worse maternal–infant bonding at 2–5 days postpartum. Some studies have investigated whether prenatal depressive symptoms predict 1-month postpartum bonding failure (Farre-Sender et al. 2018; Ohara et al. 2018). Nevertheless, no study has examined the association with bonding in the early postpartum period, such as at 25 days postpartum. Although the mechanism is unclear, the finding that prenatal depressive symptoms are associated with worse early postnatal bonding contributes to perinatal mental healthcare. By measuring prenatal depressive symptoms, it may be possible to assess the risk of bonding failure in the early postpartum period and intervene during pregnancy. Furthermore, as prenatal depressive symptoms are related to postnatal depressive symptoms (Ohara et al. 2017, 2018), the risks of both postnatal bonding failure and depressive symptoms may be assessed using prenatal depressive symptoms.

Second, prenatal anxiety was predicted to worsen maternal–infant bonding at 2–5 days and 1 month postpartum. Results of GEE also showed that prenatal anxiety was associated with postpartum MIBS scores. This result was similar to that reported by Farre-Sender et al. (2018), who found that prenatal anxiety is associated with maternal–infant bonding disturbances at 6–7 weeks postpartum. Prenatal anxiety may predict maternal–infant bonding more than depressive symptoms over the longer term.

Lower prenatal salivary oxytocin levels predicted higher MIBS scores at 2–5 days postpartum. This implies that lower oxytocin levels during pregnancy are associated with worse maternal–infant bonding. Prenatal oxytocin levels were also associated with postpartum MIBS scores even in the results of GEE. Oxytocin has been reported to be related to anxiety and mood disorders (McDonald and McDonald 2010), and is also known to be an agent in calm and connection responses (Uvnäs-Moberg et al. 2005). It is possible that prenatal oxytocin levels also reflect prenatal maternal–fetal bonding. However, this remains unclear because prenatal bonding has not yet been investigated. Additionally, to the best of our knowledge, no study has examined the association between oxytocin levels during pregnancy and pre- and postpartum bonding. Further studies are needed to investigate the mechanisms underlying this association. The results of this study suggest the importance of prenatal oxytocin levels in maternal–infant bonding. Prenatal salivary oxytocin levels may also be useful for screening bonding failure risk at 2–5 days postpartum.

Plasma cortisol levels are not associated with postnatal bonding. Song et al. (2017) also reported no association between salivary cortisol levels and maternal–infant bonding at 6 months postpartum. In contrast, Gordon et al. (2010) showed that salivary maternal cortisol levels predicted maternal–father–infant synchrony at 6 months postpartum. Maternal cortisol levels might be related to mother–father–infant synchrony, rather than to maternal–infant bonding failure. Alternatively, the small sample size might have affected our results.

At 3 months postpartum, prenatal mental health factors were not related to maternal–infant bonding. At 1 month, only prenatal anxiety was a predictor. Ohara et al. (2018) found that depressive symptoms during pregnancy did not predict bonding failure during the postpartum period. However, the error variables between depressive symptoms and bonding failure were correlated at 1 month postpartum. Furthermore, Dubber et al. (2015) reported that postnatal depressive symptoms were related to 3 months of postpartum bonding, whereas prenatal depressive symptoms were not. It is possible that maternal–infant bonding is related to postnatal mental health factors rather than prenatal factors, 1 month postpartum. Furthermore, it was reported that bed-sharing with the infant were negatively associated with mother-infant bonding between the ages of 6 weeks and 4 months (Mitchell et al. 2015). Hairston et al. (2011) have reported that infant sleep disturbance contributed independently to impaired bonding at 4 months postpartum. Two to four month postpartum, maternal-infant bonding might be influenced by the child's characteristics or parenting style rather than by the mother's mental health issues.

Lower postnatal social support predicted higher MIBS scores at all postnatal points: 2–5 days, 1 month, and 3 months postpartum. Lower postnatal social support was associated with worse maternal–infant bonding until 3 months postpartum. Similarly, previous studies have reported a relationship between social support and maternal–infant bonding (Badr et al. 2018; Kinsey et al. 2014; Ohara et al. 2018). Sufficient postpartum social support may be important for maternal–infant bonding. However, as it is difficult to use postpartum social support as a predictor of postpartum bonding, the relationship between prenatal social support and postpartum bonding needs to be examined.

This study has some limitations. First, the primiparas in this study were from a single hospital; therefore, the generalizability of the findings is limited. Additionally, some results might not be relevant, because the number of participants is smaller than the calculated number (Type II error). Some of the participants’ data could not be included in the study. Dropout reasons such as omission of the questionnaires, failure to obtain blood samples, and high oxytocin’s inter-assay coefficients of variability resulted in lower samples for this present study. Second, maternal–infant bonding was measured using only a self-reported questionnaire. Moreover, we examined maternal–infant bonding only during the 3-month postpartum period. Considering problems such as child abuse prevention, a long-term investigation is needed.

## Conclusion

Prenatal depressive symptoms, anxiety, and lower salivary oxytocin levels were predicted to worsen maternal–infant bonding 2–5 days postpartum. Prenatal anxiety also predicted that of 1 month postpartum. Additionally, prenatal anxiety and oxytocin levels were associated with postnatal maternal–infant bonding from 2–5 days to 3 months postpartum, as shown in the GEE results. Our results suggest that these prenatal factors could be used to screen for maternal-infant bonding failure. By measuring prenatal depressive symptoms, anxiety, and salivary oxytocin levels, it may be possible to assess the risk of maternal–infant bonding failure during the early postpartum period and intervene during pregnancy. Postnatal social support predicted maternal–infant bonding at all postnatal points. Sufficient postpartum social support is important for maternal–infant bonding during the 3-month postpartum period.

## Data Availability

The datasets of the current study are available from the corresponding author on reasonable request.
